# Supplementary material for: Spatiotemporal immune atlas of a clinical-grade gene-edited pig-to-human kidney xenotransplant
Source: Nat Commun. 2024 Apr 11;15:3140. doi: 10.1038/s41467-024-47454-7 (PMC11009229; doi:10.1038/s41467-024-47454-7)
Supplement: Supplementary file 1 — Supplementary Information [file 41467_2024_47454_MOESM1_ESM.pdf]

Supplementary Information

Spatiotemporal immune atlas of the first clinical-grade gene-edited pig-to-human kidney xenotransplant

Matthew D. Cheung, Rebecca Asiimwe, Elise N. Erman, Christopher F. Fucile, Shanrun Liu, Chiao-Wang Sun, Vidya Sagar Hanumanthu, Harish C. Pal, Emma D. Wright, Gelare Ghajar-Rahimi, Daniel Epstein, Babak J. Orandi, Vineeta Kumar, Douglas J. Anderson, Morgan E. Greene, Markayla Bell, Stefani Yates, Kyle H. Moore, Jennifer LaFontaine, John T. Killian Jr., Gavin Baker, Jackson Perry, Zayd Khan, Rhiannon Reed, Shawn C. Little, Alexander F. Rosenberg, James F. George, Jayme E. Locke, and Paige M. Porrett

Supplementary Figures Table of Contents:

Supplementary Figure 1. Schematic of pig-to-human xenotransplant experiment and sample collection.

Supplementary Figure 2. Hematoxylin and eosin images of biopsy sections for spatial transcriptomics and quality control metrics for single-nuclear and single-cell RNA sequencing data.

Supplementary Figure 3. Alignment of control porcine and human kidney samples to a custom porcine-human hybrid reference genome.

Supplementary Figure 4. Sequence homology between human and porcine genes in macrophage populations.

Supplementary Figure 5. Alternative mapping strategy using species-specific modified reference genomes.

Supplementary Figure 6. Validation of immune cell origin in the porcine kidney xenograft explants using the alternative mapping approach.

Supplementary Figure 7. Cell2location workflow for estimation of cell type abundance in porcine xenograft biopsies.

Supplementary Figure 8. Distribution of pig kidney parenchymal cells in the porcine kidney xenograft.

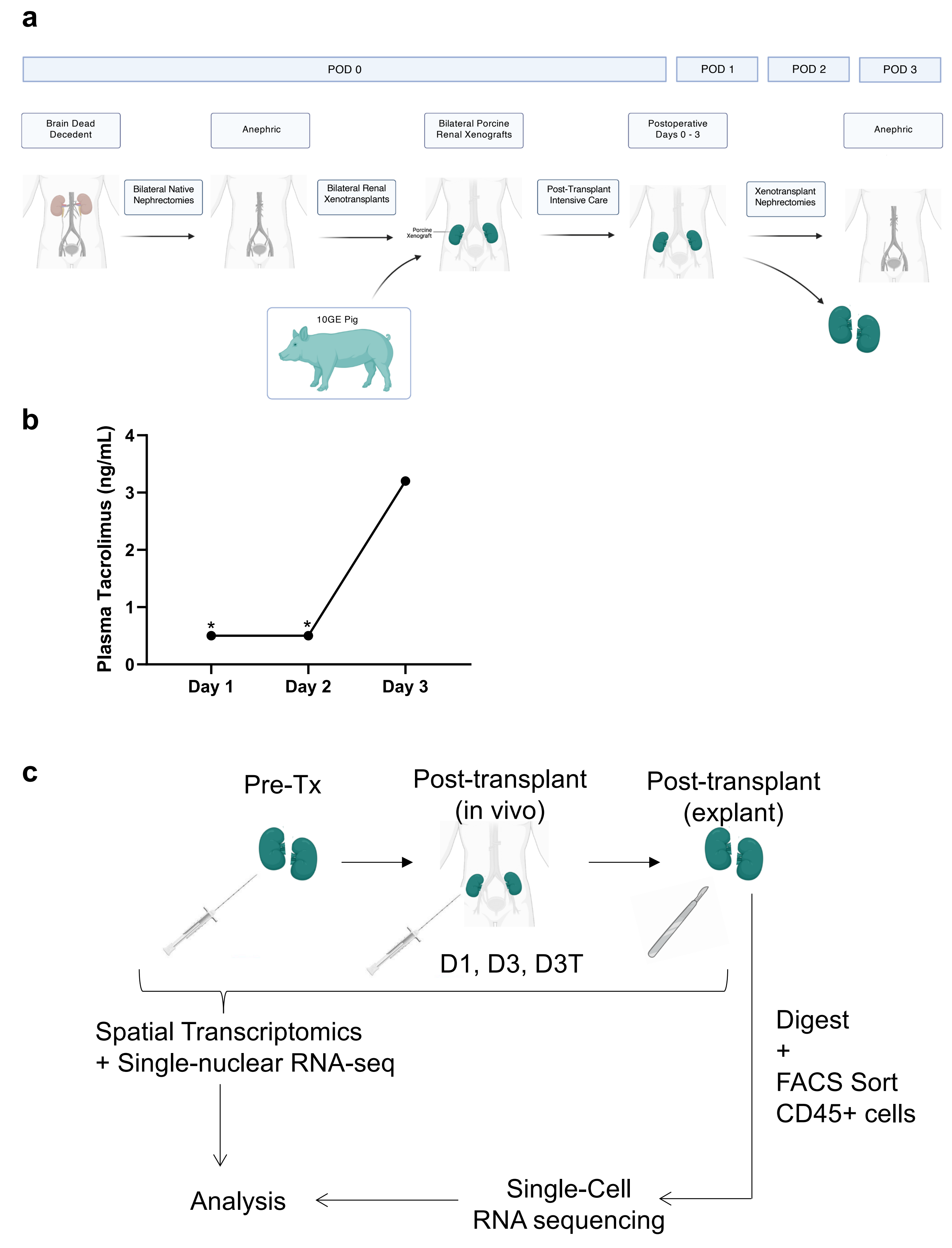

Supplementary Fig. 1

Supplementary Figure 1. Schematic of pig-to-human xenotransplant experiment and sample collection. **a**) Experimental design. Native nephrectomies were performed on a brain-dead human recipient, and bilateral porcine kidney xenografts were transplanted from a pig with 10 gene edits. Pharmacologic immunosuppression was administered from transplantation until termination approximately 74 hours later. The kidney xenografts were explanted after termination. Schematic was created with BioRender. **b)** Tacrolimus levels were measured in the clinical laboratory over the duration of the experiment. See also *Porrett et al.* 2022. *indicates tacrolimus levels reported as <2.0 ng/mL. **c)** Schematic of sample collection and wet lab workflow. Core needle biopsies were taken pre-transplant, on days 1 and 3 post-transplant, and immediately prior to termination of the experiment (i.e. day 3T). Each core biopsy was frozen in OCT and sections of the biopsy were placed on Visium slides for spatial transcriptomics. Nuclei were isolated from another section of each thawed OCT block for single-nuclear RNA-seq. Wedge biopsies were taken from the explanted xenografts and also used for single-nuclear RNA-seq. A portion of the explanted kidney xenograft representing cortex through medulla was digested to a single-cell suspension, labeled with both pig-specific and human-specific CD45 antibodies, and sorted using fluorescence activated cell sorting (FACS). Single-cell RNA-sequencing was then performed on FACS-enriched CD45+ cells from the explanted xenograft.

a

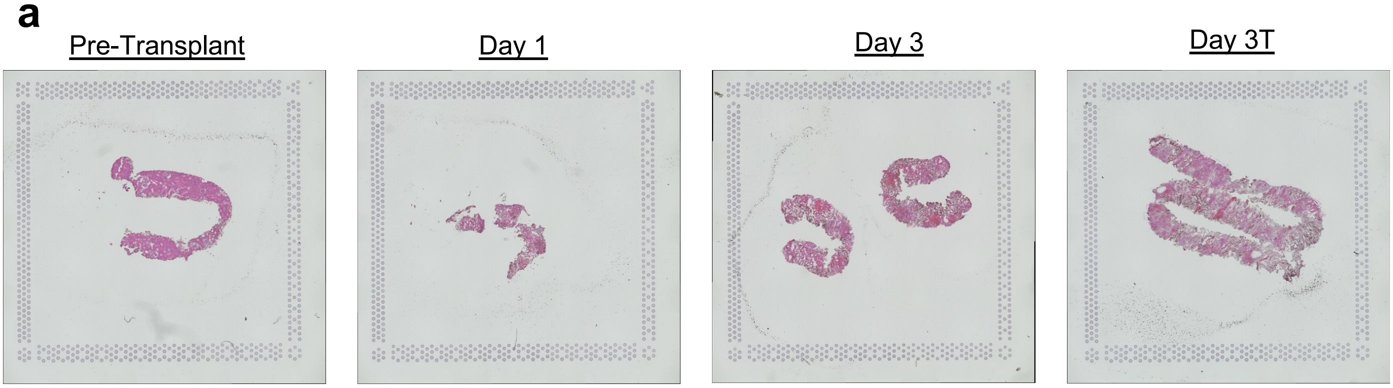

b

	Prenormalization barcoded reads	Postnormalization unique genes	Postnormalization spots
Pre-Transplant	430	10790	411
Day 1	200	5686	133
Day 3	606	12287	573
Day 3T	1057	15754	1035

c

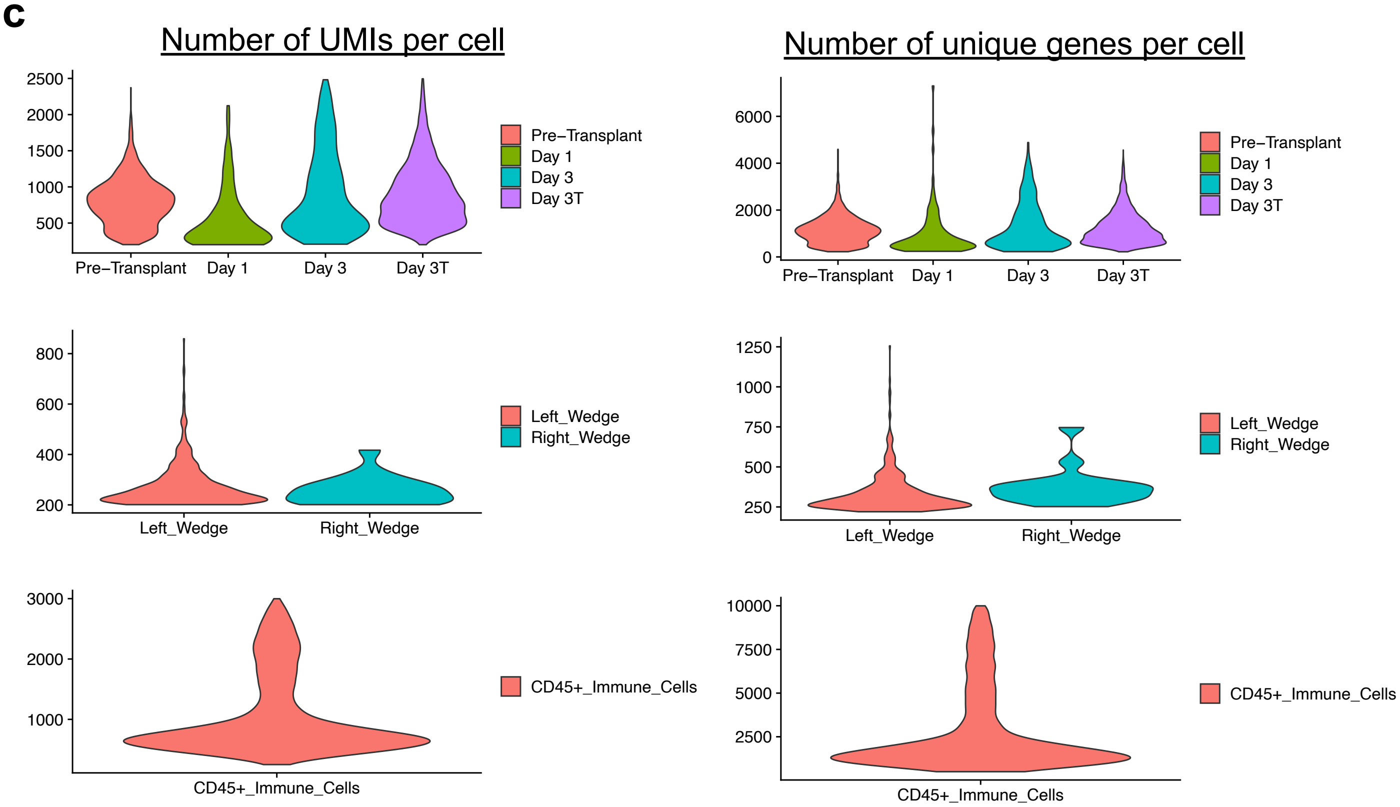

Supplementary Fig. 2

Supplementary Figure 2. Hematoxylin and eosin images of biopsy sections for spatial transcriptomics and quality control metrics for single-nuclear and single-cell RNA sequencing data. **a**) Brightfield H&E images (4X objective) taken of porcine kidney biopsy sections over the course of the experiment. Biopsies were taken from the left (Day 1 and 3T) and right (Pre-Tx and Day 3) porcine xenografts. Tissue sections are photographed after placement onto the Visium slides. **b)** Quality control metrics for each of the needle core biopsies of the porcine xenograft that were analyzed with spatial transcriptomics using the Visium gene expression platform (10X Genomics). **c)** Number of unique molecular identifiers (UMIs) and unique genes per cell for each single-nuclear (top and middle rows) or single-cell (bottom row) RNA sequencing library. Wedge biopsies (middle row) were collected from the left and right xenograft explants after termination on post-operative day three (i.e. day 3T).

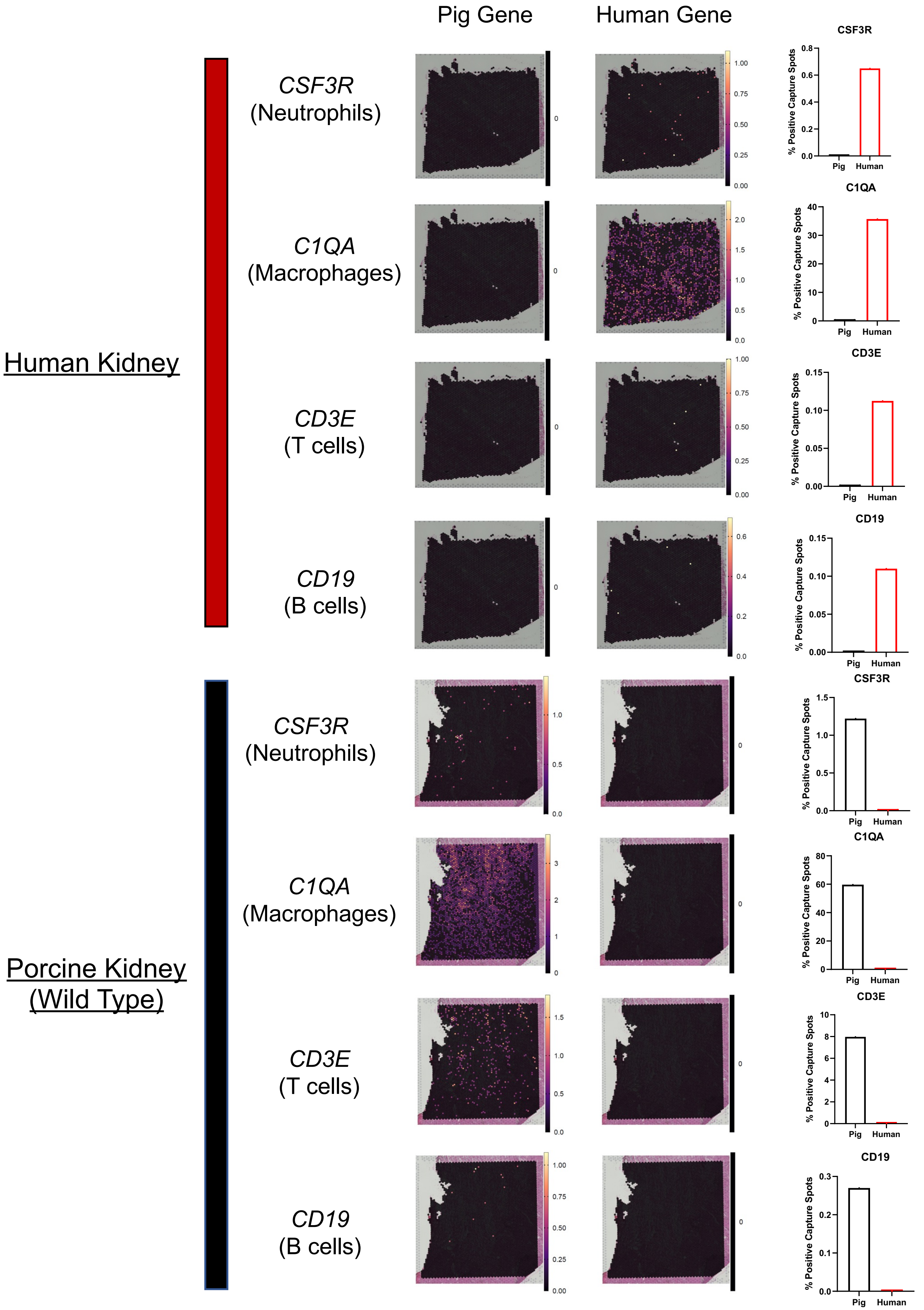

Supplementary Fig. 3

Supplementary Figure 3. Alignment of control porcine and human kidney samples to a custom porcine-human hybrid reference genome. A cortical sample of human or wild-type porcine kidney was flash frozen in optimal cutting temperature (OCT) media and a 10-μm section was placed on Visium spatial transcriptomics slides for analysis. Sequenced reads were aligned to a custom porcine-human hybrid reference genome. Read alignment of select immune cell marker genes (*CSF3R, C1QA, CD3E, and CD19*) was assessed in a species-specific fashion (i.e. reads from the porcine kidney aligned to the *ss11-CSF3R* component of the hybrid reference genome [not *hg38-CSF3R*] and vice versa). Results are quantified as a percentage of capture spots containing the gene of interest from the whole sample.

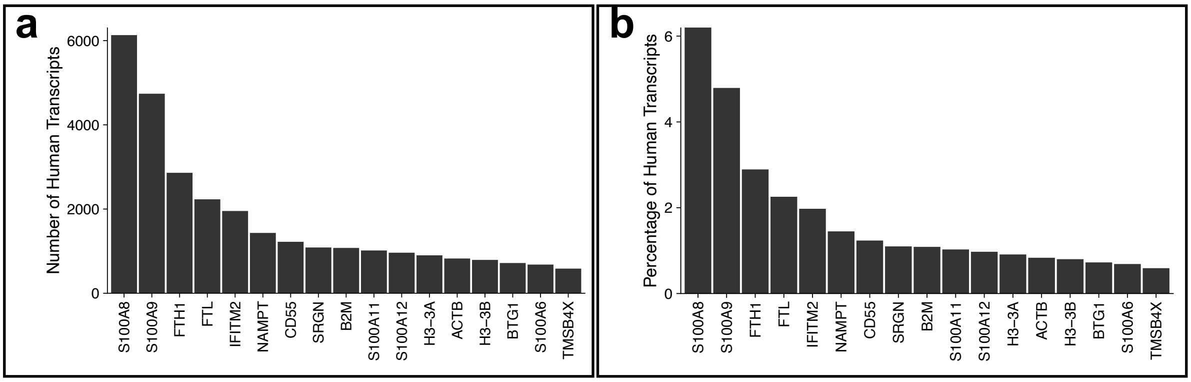

c

S100A8

80% identical

```
Query  68   TGGGGCAAGTCCGTGGGCATCATGTTGACCGAGCTGGAGAAAGCCTTGAACTCTATCATC  127
            |||| ||| ||| |||||| |||| |||| || |||||||  ||| | |||||  |||||
Sbjct  25   TGGGACAAATCCTTGGGCACCATGCTGACGGATCTGGAGAGTGCCATTAACTCCCTCATC  84

Query  128  GACGTCTACCACAAGTACTCCCTGATAAAGGGGAATTTCCATGCCGTCTACAGGGATGAC  187
            || |||| ||||||||||||||||   || ||||||| ||| ||| |||||  |||||||
Sbjct  85   GAAGTCTTCCACAAGTACTCCCTGGAGAAAGGGAATTACCACGCCATCTACGCGGATGAC  144

Query  188  CTGAAGAAATTGCTAGAGACCGAGTGTCCTCAGTATATCAGGAAAAAGGGTGCAGACGTC  247
             |||||| |||| ||||||| ||||||||| ||||||| | ||||||||  |||||   |
Sbjct  145  TTGAAGAGATTGTTAGAGACGGAGTGTCCTAAGTATATGAAGAAAAAGGACGCAGAAACC  204

Query  248  TGGTTCAAAGAGTTGGATATCAACACTGATGGTGCAGTTAACTTCCAGGAGTTCCTCATT  307
            |||||||||||| |||| |||||||  |||||||||||||||||| ||||||||||||| 
Sbjct  205  TGGTTCAAAGAGCTGGACATCAACAAGGATGGTGCAGTTAACTTCGAGGAGTTCCTCATA  264

Query  308  CTGGTGATAAAGATGGGCGTGGCAGCCCACaaaaaaaGCCATGAAGAAAGCCACAAAGAG  367
            |||||||| ||| |||||||||  ||            ||||||||| | ||||||||||
Sbjct  265  CTGGTGATCAAGGTGGGCGTGGAGGC------------CCATGAAGACATCCACAAAGAG  312

Query  368  TAGCTGAGTTACTGGGCCCAGAGGCTGGGCCCCTGGACATGTACCTGCAGAATAATAAAG  427
            |||| ||| |   |||||| | |||||||||||||||| ||| ||  |||| ||||||||
Sbjct  313  TAGCAGAGCTTTGGGGCCCCGGGGCTGGGCCCCTGGACTTGTCCCCACAGAGTAATAAAG  372

Query  428  TCATCAATACCTCA  441
            |  |  ||||||||
Sbjct  373  TAGTTGATACCTCA  386
```

d

S100A9

75% identical

```
Query  6    CTCTGTGTGGCTCCTCGGCTTTGACAGAGTGCAAGACGATGACTTGCAAAATGTCGCAGC  65
            ||||||||||||||| ||||| ||||||||||| || |||| |   | ||||||||||| 
Sbjct  73   CTCTGTGTGGCTCCTGGGCTTGGACAGAGTGCAGGAAGATGGCGGACCAAATGTCGCAGA  132

Query  66   TGGAACGCAACATAGAGACCATCATCAACACCTTCCACCAATACTCTGTGAAGCTGGGGC  125
            ||||| ||| |||||| ||||| ||||||| ||||||||| ||||| |||  ||||||| 
Sbjct  133  TGGAATGCAGCATAGAAACCATTATCAACATCTTCCACCAGTACTCGGTGCGGCTGGGGA  192

Query  126  ACCCAGACACCCTGAACCAGGGGGAATTCAAAGAGCTGGTGCGAAAAGATCTGCAAAATT  185
            |||  |||||||||||||||   ||||||||| ||||||||  |||||| |||| ||| |
Sbjct  193  ACCGGGACACCCTGAACCAGAAAGAATTCAAACAGCTGGTGAAAAAAGAGCTGCCAAACT  252

Query  186  TTCTCAAGAAGGAGAATAAGAATGAAAAGGTCATAGAACACATCATGGAGGACCTGGACA  245
            ||||||||||| |||| | | |||| || | |||| | |||||| |||| ||||||||||
Sbjct  253  TTCTCAAGAAGCAGAAGAGGGATGAGAAAGCCATAAACCACATCCTGGAAGACCTGGACA  312

Query  246  CAAATGCAGACAAGCAGCTGAGCTTCGAGGAGTTCATCATGCTGATGGCGAGGCTAACCT  305
            | ||||  |||||||||||||||||||||||||||  ||||||| |||| | ||| ||  
Sbjct  313  CTAATGTGGACAAGCAGCTGAGCTTCGAGGAGTTCTCCATGCTGGTGGCCAAGCTGACGG  372

Query  306  GGGCCTCCCACGAGAAGATGCACGAG---GGTGACGAGGGCCCTGGCCACCACCATAAGC  362
              || || |||||| |||||||| ||   |    |  |||    |||||||||||   ||
Sbjct  373  TAGCTTCTCACGAGGAGATGCACAAGACCGCCCCCCCGGGAGACGGCCACCACCACGGGC  432

Query  363  CAGGCCTCGGGGAGGGCACCCCCTAAGACCACAGT---GGCCAAGA---------TCACA  410
            ||||| ||||   | ||| |  || || ||   ||   ||||| ||          | | 
Sbjct  433  CAGGCTTCGG---GAGCAGCAGCTCAGGCCCATGTGCCGGCCAGGAGAGCCAGACCCCCG  489

Query  411  GTGGCCACGGCCACGGCCACAGTCATGG------TGGCCACGGCCACAGCCACTAATCAG  464
            | |||||||||||||||||||| |||||       ||||| |||||||||||||||||||
Sbjct  490  GGGGCCACGGCCACGGCCACAGCCATGGCGGTCACGGCCATGGCCACAGCCACTAATCAG  549

Query  465  GAGGCCAGGCCACCCTGCCTCTACCCAACCAGGGCCCCGGGGCCTGTTATGTCAAACTGT  524
            ||||||||||||  |  || ||  ||| |  ||||| || || |||   || |    || 
Sbjct  550  GAGGCCAGGCCATGC-ACCCCTGTCCACCTTGGGCCACGAGG-CTGCCGTGCCGCTTTGC  607

Query  525  CTTGGCTGTGGGGCTAGGGGCT-GGGGCCAAATAAAGTCTC  564
            ||| ||||  ||||  |||||| ||||| ||||||||||||
Sbjct  608  CTTCGCTGCAGGGCAGGGGGCTGGGGGCGAAATAAAGTCTC  648
```

Supplementary Fig. 4

Supplementary Figure 4. Sequence homology between human and porcine genes in macrophage populations. Pig macrophages were selected from Fig. 1 and the 98,884 transcripts aligning to the hg38 component of the human-porcine hybrid reference were identified (Fig. 2). **a&b**) Porcine macrophage transcripts for *S100A8* and *S100A9* were the most common transcripts which aligned to human genes. Top 11 human genes to which transcripts from porcine macrophages aligned are shown. **c&d**) BLAST analysis reveals 75-80% sequence homology between *S100A8* and *S100A9*. For *S100A8* (**c**), "query" is human transcript NM_001319198.2, and "sbjct" is porcine transcript NM_001160271.3. For *S100A9* (**d**), "query" is human transcript NM_002965.4, and "sbjct" is porcine transcript NM_001177906.1.

a

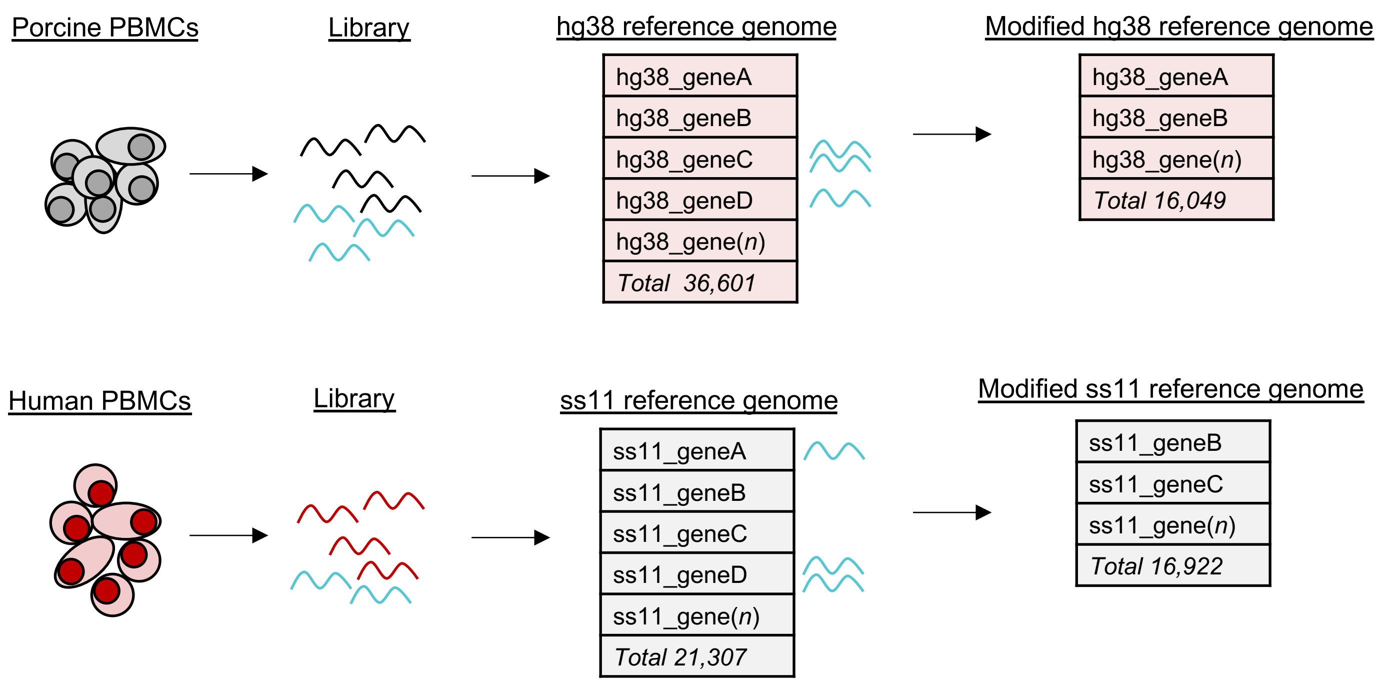

b

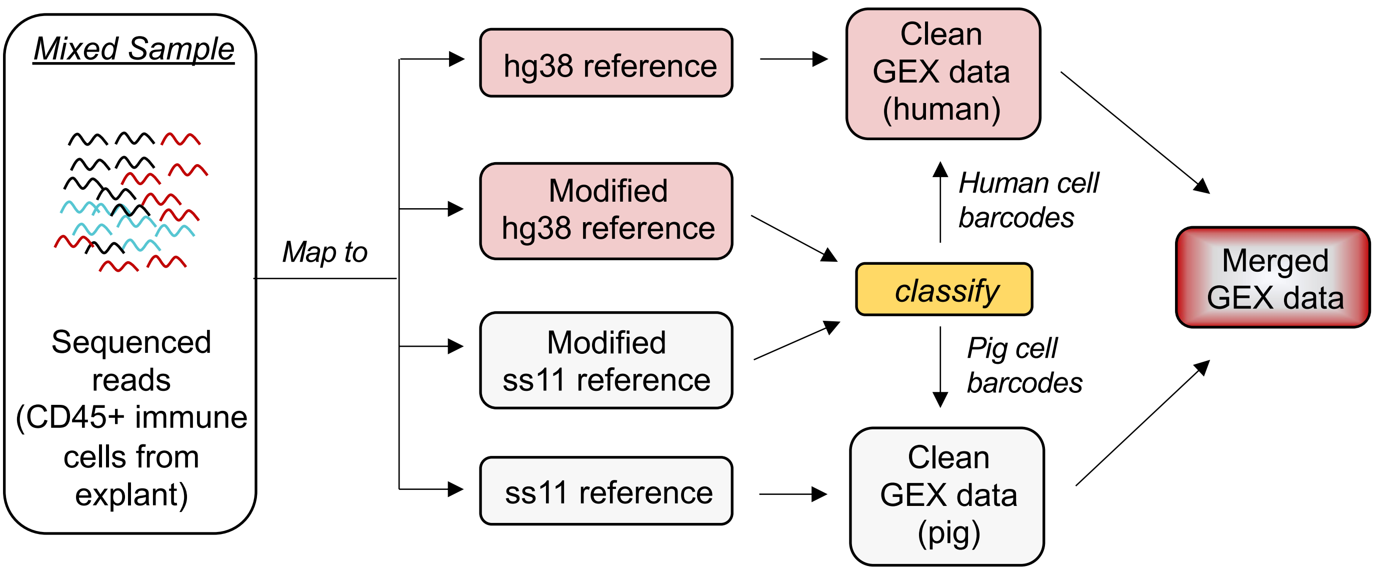

Supplementary Fig. 5

Supplementary Figure 5. Alternative mapping strategy using species-specific modified reference genomes. **a)** Conceptual schematic of construction of modified species-specific genome references. PBMCs from the 10-GE donor pig and the human recipient were collected prior to donation or transplantation, respectively. Libraries were prepared from single cells and scRNA-seq was performed. To identify homologous genes between the species which resulted in ambiguous read mapping, reads from each PBMC sample were aligned to the reference genome of the other species. Genes that mapped >3 reads were subsequently removed from the reference genome. All remaining genes from the reference genome were included in the modified reference genome for each species. Red: Human transcripts and genes. Black/grey: Pig transcripts and genes. Light blue: Transcripts which map to homologous genes in the opposite species. **b)** Analysis workflow for validation of species assignment by the hybrid reference approach using modified references. FASTQ files from sequenced reads of CD45+ immune cells sorted from the pig kidney xenograft explant were mapped to four different references during processing with Cell Ranger. Outputs from mapping to each of the four references are successively merged to generate an expression matrix for the mixed sample. Species-specific cellular barcodes in this expression matrix have been identified based on a subset of non-homologous genes in the modified references. ss11 = *sus scrofa* genome, assembly 11.1. hg38 = human genome assembly 38.

Cluster	nCells	Barcode Intersects with human classification	Barcodes Not Intersecting with hgClassification	Barcode Intersects with ssClassification
Human Neutrophil	4294	3921	373	0
Human Macrophage	1048	1044	4	0
Human Monocyte	530	530	0	0
Pig T Cell	262	0	262	262
Pig Macrophage	213	45	168	150
Pig Neutrophil	137	0	137	137
Human NK	17	17	0	0
Human B Cell	12	12	0	0

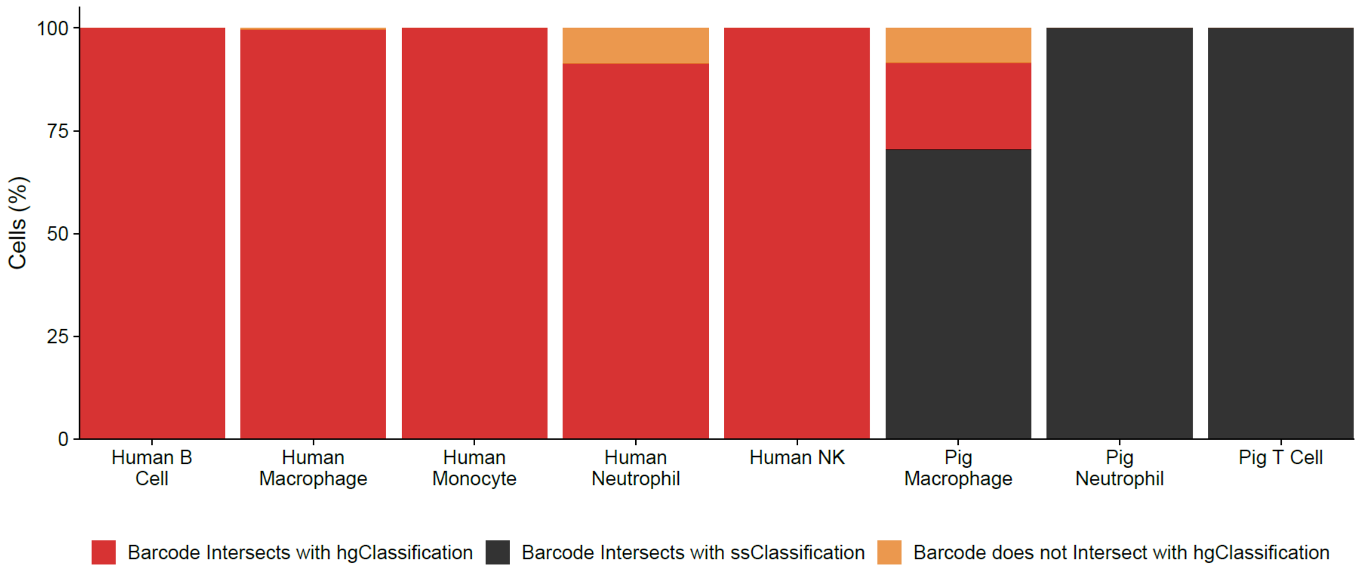

Supplementary Fig. 6

Supplementary Figure 6. Validation of immune cell origin in the porcine kidney xenograft explants using the alternative mapping approach. scRNA-seq was performed on CD45+ immune cells FACS-enriched from the explanted porcine kidney after termination (see Fig. 1 and Supplementary Fig. 1). Cells were initially identified as porcine or human in origin based on alignment against the custom human-porcine reference genome (see table column “nCells” and Fig. 1). Data were then independently processed against species-specific modified reference genomes (see Supplementary Fig. 5). Concordance between the two methods was assessed and is portrayed in the table and bar plot. Barcodes that do not intersect with either the hg38 or ss11 classifications (orange) were excluded from the modified reference alignment due to differences in data quality and filtration steps between the workflows.

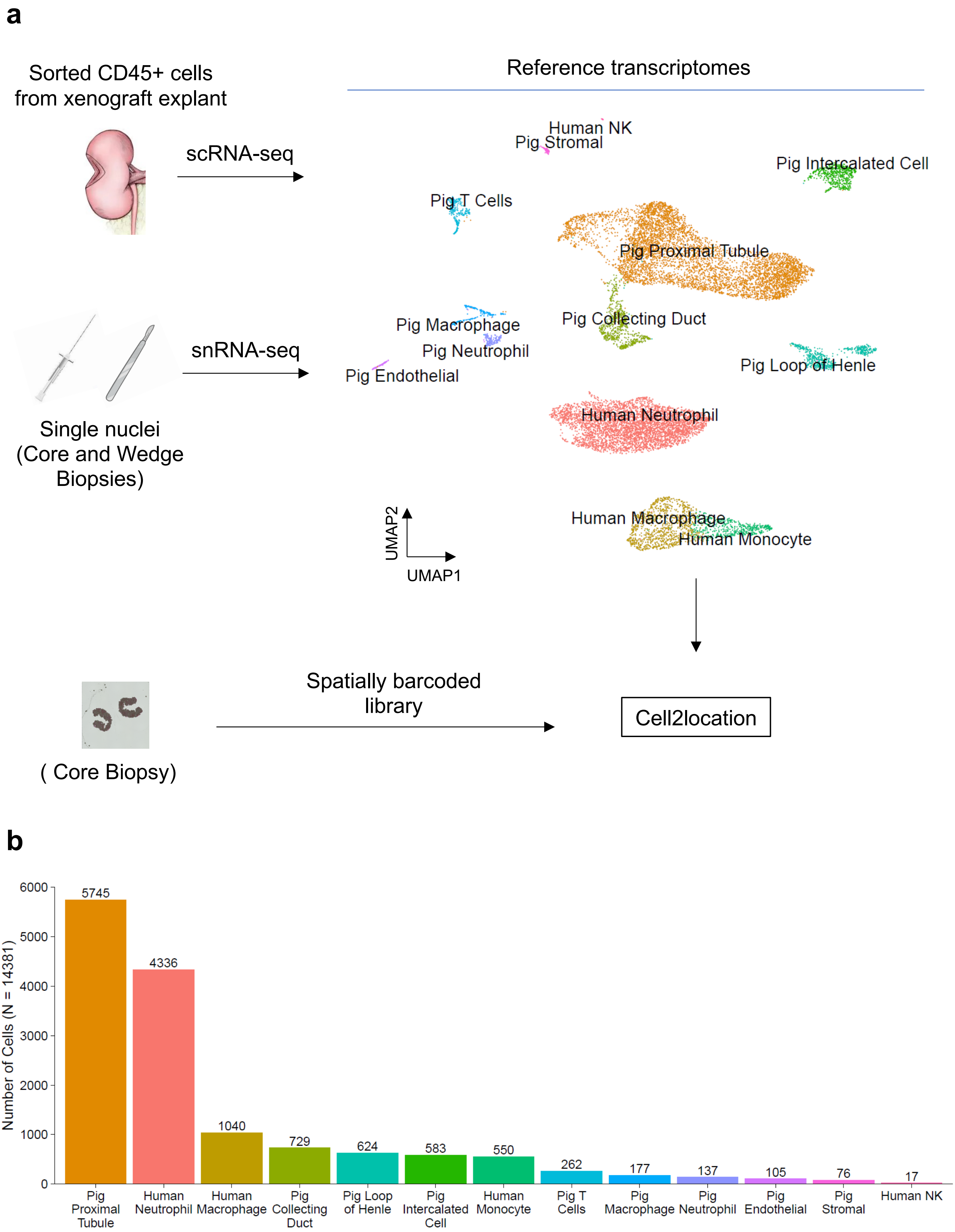

Supplementary Fig. 7

Supplementary Figure 7. Cell2location workflow for estimation of cell type abundance in porcine xenograft biopsies. **a**) Schema of Cell2location workflow, which includes input of spatially barcoded biopsy data and reference transcriptomes. Reference transcriptomes that were input into Cell2location were created from sequencing of 1) single nuclei from porcine kidney xenograft biopsies and 2) single CD45+ cells enriched from the porcine kidney xenograft after removal from the recipient (explant) (see Supplementary Fig. 1 and Fig. 1). A biopsy of the porcine kidney was taken pre-transplant and sequentially after transplant (Supplementary Fig. 1). Each biopsy was sectioned and placed on a spatial gene expression slide. The remainder of each biopsy was thawed and nuclei from the block were isolated for snRNA-seq. For generation of reference transcriptomes, all six libraries generated from the core and wedge biopsy nuclei were aggregated together with the CD45+ immune cells. UMAP of reference transcriptomes represents 14,381 cells. Schematic was created with BioRender. **b**) Enumeration of reference transcriptomes represented in UMAP in (**a**).

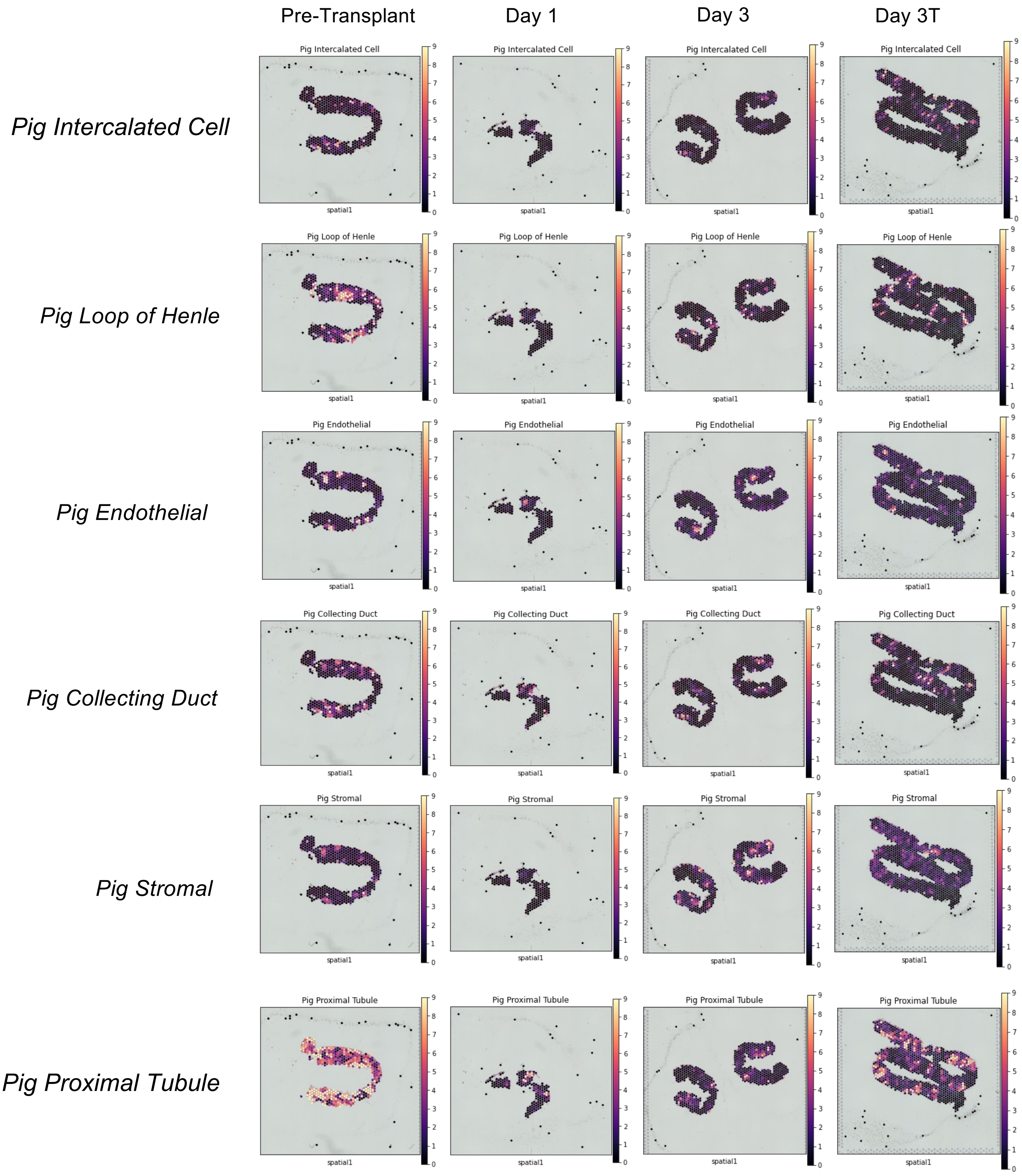

Supplementary Fig. 8

Supplementary Figure 8. Distribution of pig kidney parenchymal cells in the porcine kidney xenograft. As in Fig. 3, spatial transcriptomics was performed on serial needle core biopsies of 10-GE porcine kidneys before and after transplantation into a brain-dead human recipient. Cell type signatures were identified from reference transcriptomes using Cell2location and used to determine the distribution of pig kidney parenchymal cells throughout the biopsies.
“Pre-tx” = pre-transplant. Day 3T biopsy was taken on post-transplant day 3 at study termination.
