# Supplementary material for: Spatiotemporal immune atlas of a clinical-grade gene-edited pig-to-human kidney xenotransplant
Source: Nat Commun. 2024 Apr 11;15:3140. doi: 10.1038/s41467-024-47454-7 (PMC11009229; doi:10.1038/s41467-024-47454-7)
Supplement: Supplementary file 3 — Description of Additional Supplementary Files [file 41467_2024_47454_MOESM3_ESM.pdf]

Description of Additional Supplementary Files:

Supplementary Data 1: Differentially expressed genes for each respective cell-type.

Differential expression (DE) analysis between clusters/two groups of cells was performed using the non-parametric Wilcoxon Rank Sum test implemented in the FindMarkers() functions from the Seurat R package. DE testing was limited to genes which show, on average, at least 0.1 log-fold change between the two groups of cells and on genes that are detected in a minimum fraction of 0.01 cells in either of the two populations. A gene was defined as differentially expressed if the absolute average log fold-change (avg_logFC) was >0.25 and the Bonferroni-adjusted p-value <0.01.

Supplementary Data 2: List of genes used for macrophage phenotyping in Figure 5

Genelist_m1: list of pro-inflammatory ("M1-like") genes adapted from Xue *et al*. (manuscript reference 26)

Genelist_m2: list of anti-inflammatory ("M2-like") adapted from Xue *et al*. (manuscript reference 26)
