# Supplementary material for: Spatiotemporal immune atlas of a clinical-grade gene-edited pig-to-human kidney xenotransplant
Source: Nat Commun. 2024 Apr 11;15:3140. doi: 10.1038/s41467-024-47454-7 (PMC11009229; doi:10.1038/s41467-024-47454-7)
Supplement: Supplementary file 6 — Reporting Summary [file 41467_2024_47454_MOESM6_ESM.pdf]

nature portfolio

Corresponding author(s): Paige M. Porrett

Last updated by author(s): March 20 2024

Reporting Summary

Nature Portfolio wishes to improve the reproducibility of the work that we publish. This form provides structure for consistency and transparency in reporting. For further information on Nature Portfolio policies, see our Editorial Policies and the Editorial Policy Checklist.

Statistics

For all statistical analyses, confirm that the following items are present in the figure legend, table legend, main text, or Methods section.

n/a	Confirmed	
☐	☒	The exact sample size (*n*) for each experimental group/condition, given as a discrete number and unit of measurement
☐	☒	A statement on whether measurements were taken from distinct samples or whether the same sample was measured repeatedly
☐	☒	The statistical test(s) used AND whether they are one- or two-sided<br>*Only common tests should be described solely by name; describe more complex techniques in the Methods section.*
☐	☒	A description of all covariates tested
☐	☒	A description of any assumptions or corrections, such as tests of normality and adjustment for multiple comparisons
☐	☒	A full description of the statistical parameters including central tendency (e.g. means) or other basic estimates (e.g. regression coefficient) AND variation (e.g. standard deviation) or associated estimates of uncertainty (e.g. confidence intervals)
☐	☒	For null hypothesis testing, the test statistic (e.g. *F*, *t*, *r*) with confidence intervals, effect sizes, degrees of freedom and *P* value noted<br>*Give P values as exact values whenever suitable.*
☒	☐	For Bayesian analysis, information on the choice of priors and Markov chain Monte Carlo settings
☒	☐	For hierarchical and complex designs, identification of the appropriate level for tests and full reporting of outcomes
☒	☐	Estimates of effect sizes (e.g. Cohen's *d*, Pearson's *r*), indicating how they were calculated

Our web collection on statistics for biologists contains articles on many of the points above.

Software and code

Policy information about availability of computer code

Data collection	Publicly available packages and pipelines were used to process FASTQ files from scRNA-seq, snRNA-seq, and spatial transcriptomics experiments. These include Cell Ranger (6.1.1), Space Ranger (1.3). Use of these packages is described in detail in the Methods section.
Data analysis	Data analysis was performed with R (4.2.1) or python (3.9) using open source software packages: Seurat (4.2.0), SoupX (1.6.1), Harmony (0.1.0), Scanpy (1.9), Cell2location (0.1). Code used in this manuscript is available on GitHub at https://github.com/PorrettLab/Spatiotemporal-immune-atlas-of-the-1st-clinical-grade-gene-edited-pig-to-human-kidney-xenotransplant/tree/main (https://zenodo.org/records/10809481)

For manuscripts utilizing custom algorithms or software that are central to the research but not yet described in published literature, software must be made available to editors and reviewers. We strongly encourage code deposition in a community repository (e.g. GitHub). See the Nature Portfolio guidelines for submitting code & software for further information.

Data

Policy information about availability of data

All manuscripts must include a data availability statement. This statement should provide the following information, where applicable:

- Accession codes, unique identifiers, or web links for publicly available datasets
- A description of any restrictions on data availability
- For clinical datasets or third party data, please ensure that the statement adheres to our policy

The human and wild-type control pig kidney data generated in this study have been deposited in the Gene Expression Omnibus database under accession code GSE242270. The data from the 10-GE pig have been deposited to the European Genome-Phenome Archive (EGA) Database under study accession code

EGAS50000000244 and data accession code EGAD50000000359 and are available under restricted access due to privacy restrictions given the proprietary nature of the 10-gene edited porcine kidney product. Data access can be obtained by reaching out via email to the corresponding author (pmporrett@uabmc.edu) who will provide instructions within one week to the requestor so that the requestor may apply for data access from the EGA. In brief, the requestor will need to review and agree to the conditions of the EGA Data Access Agreement. Source data are provided as a Source Data file.

Research involving human participants, their data, or biological material

Policy information about studies with human participants or human data. See also policy information about sex, gender (identity/presentation), and sexual orientation and race, ethnicity and racism.

Reporting on sex and gender	One male human decedent was used in this study. The sex of the decedent in reported in the Methods.
Reporting on race, ethnicity, or other socially relevant groupings	n/a
Population characteristics	57-year-old brain-dead male who died of blunt trauma
Recruitment	The participant was recruited into the study based on his status as a brain-dead individual whose organs could not be used for transplantation. Additional criteria for study recruitment is included in a related publication (Porrett et al., AJT 2022) and is cited in the manuscript.
Ethics oversight	Institutional Research Board approval was obtained from the University of Alabama at Birmingham, although decedents are not considered human subjects research. Nevertheless, this oversight was sought by investigators given the unique nature of the study. The IRB is reported in the manuscript: IRB-300004648.

Note that full information on the approval of the study protocol must also be provided in the manuscript.

Field-specific reporting

Please select the one below that is the best fit for your research. If you are not sure, read the appropriate sections before making your selection.

☒ Life sciences ☐ Behavioural & social sciences ☐ Ecological, evolutionary & environmental sciences

For a reference copy of the document with all sections, see nature.com/documents/nr-reporting-summary-flat.pdf

Life sciences study design

All studies must disclose on these points even when the disclosure is negative.

Sample size	Sample size of n=1 is reported in the manuscript. Only one decedent was available for study given the proof-of-concept nature of this study, constraints around recruitment of the study and eligibility requirements, as well as financial constraints.
Data exclusions	No data were excluded from analysis.
Replication	There was no opportunity to replicate the study with another decedent given the constraints described under "sample size" above. Serial biopsies of the kidney xenografts were performed, thus allowing longitudinal replicates.
Randomization	n/a. There is no control decedent in the study that did not receive immunosuppression or a non-gene modified kidney xenotransplant given the nature of the study design (see also "sample size" above). Immunosuppression is required in allotransplantation to avoid rapid rejection, and ample preclinical data provided the rationale for humanization of the kidney.
Blinding	Blinding is not applicable given there was only one recipient in this study.

Reporting for specific materials, systems and methods

We require information from authors about some types of materials, experimental systems and methods used in many studies. Here, indicate whether each material, system or method listed is relevant to your study. If you are not sure if a list item applies to your research, read the appropriate section before selecting a response.

Materials & experimental systems

n/a	Involved in the study	
☐	☒	Antibodies
☒	☐	Eukaryotic cell lines
☒	☐	Palaeontology and archaeology
☐	☒	Animals and other organisms
☒	☐	Clinical data
☒	☐	Dual use research of concern
☒	☐	Plants

Methods

n/a	Involved in the study	
☒	☐	ChIP-seq
☒	☐	Flow cytometry
☒	☐	MRI-based neuroimaging

Antibodies

Antibodies used	5uL per test of 0.5 mg per mL anti-human CD45 FITC clone 2D1 (Biolegend Inc., Cat. No. 368508) and 10 µL per 100 µL cell suspension of mouse anti-pig CD45-Alexa Fluor® 647 conjugate antibody clone K252.1E4 (Bio-Rad Laboratories, Inc, Cat. No. MCA1222A647)
Validation	All antibodies used are commercially available and have been validated by the producer for flow cytometry on human and porcine cells:<br>FITC: Anti-human CD45; https://www.biolegend.com/en-gb/products/fitc-anti-human-cd45-antibody-12394?GroupID=BLG14850<br>AF 647 anti-pig CD45; https://www.bio-rad-antibodies.com/monoclonal/pig-porcine-cd45-antibody-k252-1e4-mca1222.html?f=alexa%20fluor%C2%AE%20647

Animals and other research organisms

Policy information about studies involving animals; ARRIVE guidelines recommended for reporting animal research, and Sex and Gender in Research

Laboratory animals	A male Chester-White crossbreed, 368 days old, 159 kgs, was used as the porcine kidney donor. This donor was maintained in a designated pathogen-free facility near the UAB campus.
Wild animals	No wild animals were used in the study.
Reporting on sex	Animal sex is not reported in this manuscript but was reported in a prior publication relevant to this study (Porrett et al., Amer J Transpl 2022)
Field-collected samples	No field collected animals were used in the study.
Ethics oversight	The study was approved by the University of Alabama at Birmingham IACUC (IACUC-22015), which is reported in the manuscript.

Note that full information on the approval of the study protocol must also be provided in the manuscript.
